# Combination of Phenethyl Isothiocyanate and Dasatinib Inhibits Hepatocellular Carcinoma Metastatic Potential through FAK/STAT3/Cadherin Signalling and Reduction of VEGF Secretion

**DOI:** 10.3390/pharmaceutics15102390

**Published:** 2023-09-27

**Authors:** Gabriele Strusi, Caterina M. Suelzu, Shannon Weldon, Jennifer Giffin, Andrea E. Münsterberg, Yongping Bao

**Affiliations:** 1 Norwich Medical School, University of East Anglia, Norwich NR4 7TJ, UK; 2 School of Biological Sciences, University of East Anglia, Norwich NR4 7TJ, UKa.munsterberg@uea.ac.uk (A.E.M.)

**Keywords:** combination therapy, PEITC, dasatinib, oncology, cancer therapeutics, drug development, FAK, VEGF, HCC, CAM assay

## Abstract

Cancerous cells are characterised by their ability to invade, metastasise, and induce angiogenesis. Tumour cells use various molecules that can be targeted to reverse these processes. Dasatinib, a potent Src inhibitor, has shown promising results in treating hepatocellular carcinoma (HCC) in vitro and in vivo. However, its effectiveness is limited by focal adhesion kinase (FAK) activation. Isothiocyanates, on the other hand, are phytochemicals with broad anticancer activity and FAK inhibition capabilities. This study evaluated the synergistic effect of dasatinib and phenethyl isothiocyanate (PEITC) on HCC. The combination was tested using various assays, including MTT, adhesion, scratch, Boyden chamber, chorioallantoic membrane (CAM), and yolk sac membrane (YSM) assays to evaluate the effect of the drug combination on HCC metastatic potential and angiogenesis in vitro and in vivo. The results showed that the combination inhibited the adhesion, migration, and invasion of HepG2 cells and reduced xenograft volume in the CAM assay. Additionally, the combination reduced angiogenesis in vitro, diminishing the growth of vessels in the tube formation assay. The inhibition of FAK/STAT3 signalling led to increased E-cadherin expression and reduced VEGF secretion, reducing HCC metastatic potential. Therefore, a combination of PEITC and dasatinib could be a potential therapeutic strategy for the treatment of HCC.

## 1. Introduction

Hepatocellular carcinoma (HCC) is the most common type of liver cancer; at advanced stages, the five-year survival rate is only 20% [1]. Diagnosis at early stages is difficult to achieve, and usually, affected patients are usually diagnosed at advanced stages of the disease. The most common treatment for HCC is the systemic administration of sorafenib, which has been the only treatment approved for more than a decade [2]. Over the last five years, several new drugs have been approved for the treatment of HCC, but the rate of FDA approval for new HCC treatments is among the lowest when compared with other types of cancer [3]. Therefore, there is an urgent need for novel treatment options for HCC.

Dasatinib is a potent multi-targeted inhibitor used for the treatment of chronic myeloid leukaemia and it has also been evaluated for the treatment of solid tumours [4]. Dasatinib showed promising results in reducing cell proliferation in several HCC cell lines [5,6]. However, its potential in vivo was shown to be limited by the activation of the focal adhesion kinase (FAK) pathway and improved with association with FAK inhibitors [7]. Furthermore, dasatinib showed improved cytotoxicity towards HCC cells when combined with other agents [8,9,10], with particular potential in sorafenib-resistant tumours [6].

Isothiocyanates (ITCs) are compounds known for their ability to inhibit FAK activation. ITCs derive from glucosinolates, phytochemicals abundant in cruciferous vegetables [11]. Epidemiological studies showed an inverse association between the consumption of this class of vegetables and the risk of cancer development, underlying the relevance of ITCs in cancer research [12]. Indeed, numerous reports showed the role of ITCs in reducing HCC growth [13] and HCC metastatic potential [14]. Moreover, it has been demonstrated that ITCs can improve the anticancer activity of other approved chemotherapeutic agents, repurposing their use in non-small cell lung cancer [15], breast cancer [16], and gastric cancer [17].

Recent clinical trials pointed at the combination of different drugs as the most successful approach in the treatment of HCC [2]. The combination of ITCs and dasatinib has not yet been assessed for the treatment of solid tumours. Here, we tested whether the anticancer potential of ITCs can improve the action of dasatinib for the treatment of HCC. In preliminary screening, the efficiency of different ITCs combined with dasatinib was tested in HepG2 cells. The addition of phenethyl isothiocyanate (PEITC) to dasatinib treatment showed the most promising result with a synergistic increase in cytotoxicity towards HepG2 cells.

This study evaluated the PEITC and dasatinib combination, termed PDc, to determine its ability to reduce the HCC metastatic potential. The results showed that PDc possesses an improved synergistic effect towards HCC HepG2 cells in vitro and chick embryos assays in vivo, reducing the metastatic potential and secretion of pro-angiogenic factors through the multiple targeting of the Src/FAK pathway.

## 2. Materials and Methods

Specific of the products (i.e., catalogue numbers) are provided in the Supplementary Materials Table S1.

### 2.1. Cell Lines

HepG2 cells (HB-8065, American Type Culture Collection, Manassas, VA, USA) were cultured in DMEM supplemented with 10% FBS, 1% L-glutamine (200 mM), penicillin (100 U/mL), and streptomycin (100 µg/mL), at 37 °C, 5% (*v*/*v*) CO_2_. HUVECs cells (C-12200, PromoCell GmbH, Heidelberg, Germany) were cultured in EGM2 supplemented with Supplement Mix/Endothelial (PromoCell GmbH, Heidelberg, Germany), penicillin (100 U/mL) and streptomycin (100 µg/mL), at 37 °C, 5% (*v*/*v*) CO_2_. HUVECs were cultured in flasks pre coated with 2% collagen solution (4 mg/mL) to a maximum of 8 passages as specified by the supplier (15 doublings). Hepa 1–6 cells (CRL-1830, American Type Culture Collection, Manassas, Virginia, USA) were cultured in DMEM supplemented with 10% FBS, 1% L-glutamine (200 mM), penicillin (100 U/mL), and streptomycin (100 µg/mL), at 37 °C, 5% (*v*/*v*) CO_2_.

### 2.2. Cell Viability Assay

Cells (HepG2 7 × 10^3^ cells/well, HUVECs 5 × 10^3^ cells/well) were seeded in 96-well plates and received the drug treatment once 80% confluent. After 24 h, 100 µL of 10% 5 mg/mL MTT in DMEM were added to each well and incubated at 37 °C for 1 h. Formazan crystals were then dissolved in 100 µL of DMSO per well. The absorbance was determined using a FLUOstar omega plate reader at 550 nm. Combination index (CI) values was calculated using Compusyn software (ComboSyn Inc., Paramus, NJ, USA).

### 2.3. Cell Adhesion Assay

96-well plates were coated with 50 µL of 2% Matrigel^®^ (10 mg/mL) (Corning, Somerville, MA, USA). Cells (HepG2, HUVEC 1 × 10^5^ cells/well) were seeded with FBS-free DMEM added with test compounds. After 3 h, medium was removed, and the wells were washed three times with PBS to remove non-adhered cells. Cells were fixed with 10% buffered formalin for 20 min. After that, cells were stained with 0.1% crystal violet for 20 min. Finally, the wells were washed three times and single wells were imaged with a Leica DMI3000 B (Leica Biosystems, Nußloch, Germany). Quantification of adhesion rate was calculated using ImageJ 1.53k software to quantify the area occupied by adhered cells.

### 2.4. Scratch Wound Healing Assay

HepG2 cells (2.5 × 10^5^ cells/well) were seeded in 24-well plates and once 100% of confluence was reached, scratches were made with a 200 µL pipette tip across the centre of the well. Wells were then washed twice with 1% FBS medium and then DMEM 1% FBS was added containing test compounds. Wells were imaged at 4 experimental times, 0 h, 24 h, 48 h, and 72 h. Three pictures were taken from each well using a Leica DMI3000 B microscope with a 5× magnification. Pictures were taken from the same spot determined by a permanent marker sign at the bottom of the plate. The wound area was evaluated using ImageJ 1.53k software with the macro “MRI Wound Healing Tool” described in Cormier et al. [18].

### 2.5. Migration/Invasion Boyden Chamber Assays

Cells (HepG2 1 × 10^5^, HUVECs 2 × 10^5^) were seeded on the top chamber of the Boyden chamber ThinCert™ 8 µm in FBS-free DMEM with addition of test compounds. Lower chamber was filled with 500 µL of DMEM 10% FBS. After 48 h, non-migrated cells were removed using a cotton swab. The inserts were washed twice with PBS and fixed with 10% buffered formalin for 20 min. Subsequently, the inserts were washed twice with PBS and the migrated cells stained with 0.1% crystal violet to stain them for 20 min. Finally, the inserts were washed twice with dH_2_O and left in a fume hood to air dry. Inserts were imaged using a Leica DMI3000 B microscope and 5 pictures were taken from each insert from random fields. Migrated/invaded cells were counted by an independent operator. The invasion assay was performed as the migration assay but with a coating of 1% Matrigel^®^ (10 mg/mL) in FBS-free DMEM in the top chamber.

### 2.6. CAM In Ovo Tumour Xenograft Assay

The CAM assay is adapted from Kunz P. et al., 2019 with some modifications [19]. Fertilised Shaver Brown chicken eggs were incubated at 37 °C. On day 1 (ED1), 6 mL albumen were removed with a syringe. Eggs were incubated in a horizontal positioning until ED7. One day prior to xenografting, 40 µL of Cultrex Basement Membrane Extract, Type 3 mixed with vehicle or drug treatment with 1 × 10^6^ HepG2, were pipetted on a Petri dish and incubated at 37 °C to solidify for 1 h and then covered with DMEM^+^ and incubated overnight. On ED7, the side of the egg was covered with Leukosilk tape and a window of 1.5 cm was opened into the eggshell. A plastic ring of 8 mm internal diameter and 11 mm external diameter was placed onto the CAM and the area within was lacerated with a 30-gauge needle. The pre-formed cell pellet was placed onto the CAM delimited by the plastic ring. The eggshell window was sealed with Leukosilk and the eggs incubated for further 7 days at 37 °C. On ED14, the eggs were opened, and the developed xenograft tumours were resected and measured. The tumour relative volume ($V_{r}$) was calculated as follows:

(1) $$r_{t}=\frac{1}{2}\sqrt{D_{1}+D_{2}}$$

(2) $$V_{r}=\frac{4}{3}\times \pi \times r_{t}^{3}$$

where $r_{t}$ is the radius calculate using the two perpendicular diameters $D_{1}$ and $D_{2}$. The results presented are the mean of a minimum of three biological replicates with representative images. All experiments were performed on chicken embryos younger than 14 days of development and therefore were not regulated by the Animal Scientific Procedures Act 1986.

### 2.7. Conditioned Medium (CM)

HepG2 cells (1 × 10^6^) were seeded in 15 cm dishes and, once confluence was reached, were washed with FBS-free DMEM. Control, PEITC 12 μM, dasatinib 1.25 μM, and their combination were added to the plates in 30 mL FBS-free DMEM. The conditioned medium from each plate was collected and centrifuged at 1000× *g* 10 min to remove cells. The supernatant was collected and concentrated with a 0.45 µM filter and added to Amicon^®^ Ultra-15 3 kDa Centrifugal Filter Unit (Merck Life Science, Gillingham, UK). Filter tubes were centrifuged at 4 °C for 3000× *g* at steps of 30 min until the volume reached 500 µL (~30× concentration). Samples were aliquoted, quantified using Bradford assay, and stored at −80 °C. Conditioned medium was then diluted with EGM2^+^ prior HUVECs treatment for different assays or subjected to analysis using the Proteome Profiler Human Angiogenesis Array Kit (Bio-Techne Ltd., Abingdon, UK).

### 2.8. Angiogenesis Proteome Profiler

To identify the factors involved in the PDc anti-angenic action, a Proteome Profiler^TM^ Human Angiogenesis Antibody Array (Bio-Techne Ltd., Abingdon, UK) was employed. As per manufacturer instructions, the membranes were blocked with blocking buffer for 1 h at room temperature. In the meantime, 500 µL of diluted CM (300 µg) and the antibody detection cocktail were incubated for 1 h at room temperature. The membranes were incubated with the sample/antibody detection solution over night at 4 °C on a rocking platform. After that, the membranes were washed three times 10 min each and incubated with IRDye^®^ 800CW Streptavidin (LI-COR Biotechnology, Cambridge, UK) for 30 min at room temperature on a rocking platform. After 3 further washes of 10 min each, the membranes were imaged using the Odyssey CLx Imaging System (LI-COR Biotechnology, Cambridge, UK). The dot density was quantified using Image Studio Lite 5.2.5 software and compared with the signal of a control membrane.

### 2.9. Tube Formation Assay

96-well plates were added with 50 µL of 10 mg/mL Matrigel^®^ (10 mg/mL) (Corning, Somerville, MA, USA) and incubated at 37 °C for 40 min. HUVECs cells were harvested and resuspended at 2 × 10^5^ cells/mL, 100 µL of cell solution were added with CM diluted in EGM2 for a final volume of 125 µL, and 12.5 µg of protein loaded onto the gelified Matrigel^®^ (Corning, Somerville, MA, USA). After 16 h, the tubular structures were stained with a solution of 8 µg/mL Calcein AM in PBS. The media were removed, and the gels were washed once with PBS and then 50 µL of staining solution was added. The plates were incubated for 30 min at 37 °C and then the staining solution was removed, and the wells were washed once with PBS. Single wells were imaged using the EVOS M5000 Imaging System (ThermoFisher Scientific, Loughborough, UK) with a 470/525 nm light filter at 4× magnification. The images were analysed using ImageJ 1.53k software with the macro Angiogenesis Analyzer described in Carpentier et al. 2020 [20].

### 2.10. Ex Ovo Angiogenesis In Vivo Assay

The YSM assay is adapted from Wang et al., 2018 with some modifications [21]. Fertilised Shaver Brown chicken eggs were incubated at 37 °C for 2.5 days. Eggs were cracked opened, and their content was carefully placed on a paper boat previously sterilised. A teflon ring of 10 mm internal diameter was placed onto the yolk sac membrane, on top of a main branch of blood vessels, and 20 µL of CM 2.5 mg/mL were pipetted inside. The egg in boat was covered with a Petri dish lid and incubated at 37 °C for 36 h. Pictures were taken every 12 h to check blood vessel development using a stereomicroscope Stemi SV11. The developed blood vessels at 36 h were classified as primary, secondary, tertiary, and quaternary and quantified as described in Datar P. et al. [22]. The results presented are the mean of a minimum of three biological replicates with representative images. All experiments were performed on chicken embryos younger than 14 days of development and therefore were not regulated by the Animal Scientific Procedures Act 1986.

### 2.11. ELISA Assay

DuoSet^®^ ELISA VEGF kit (Bio-Techne Ltd., Abingdon, UK) was employed to evaluate VEGF levels in non-concentrated CM samples following manufactures instructions. Absorbance was read at 450 nm with a FLUOstar omega plate reader. The generated standard curves were used to establish the samples analyte concentrations considering a 3× sample dilution.

### 2.12. Western Blotting

Protein content was isolated from treated cells using a solution of 20 mM Tris-HCl (pH 8), 150 mM NaCl, 2 mM EDTA, 10% glycerol, and 1% Nonidet P40, added with Protease Inhibitor Cocktail (Cell Signaling Technologies, Leiden, The Netherlands) and Pierce™ Phosphatase Inhibitor Mini Tablets (ThermoFisher Scientific, Loughborough, UK). Protein content was quantified using Bradford reagent (Merck Life Science, Gillingham, UK) and a FLUOstar omega plate reader at 595 nm. An average of 20–40 µg of protein sample was loaded onto SDS-polyacrylamide gels and separated in a Mini Protean Tank filled with TruPAGE™ TEA-Tricine SDS Running Buffer (Merck Life Science, Gillingham, UK). Molecular weight was determined using PageRuler™ Plus (Invitrogen, ThermoFisher Scientific, Loughborough, UK). Proteins were transferred into a PVDF membrane using a semi dry Trans-Blot^®^ Turbo™ Transfer System (Bio-Rad Laboratories, Hercules, CA, USA). Protein blocking was performed using Intercept TBS blocking buffer and target detection was achieved with incubation with primary antibody solution overnight at 4 °C. After washing, target detection was achieved with incubation with a secondary antibody IRDye^®^ solution (LI-COR Biotechnology, Cambridge, UK) for 1 h on low rocker speed at room temperature. After the final wash membranes were scanned with an Odyssey CLx Imaging System (LI-COR Biotechnology, Cambridge, UK). The protein band density was quantified using Image Studio Lite 5.2.5 software and normalised to the signal of β-actin. Details of antibodies used are provided in Supplementary Materials Table S2.

### 2.13. Immunocytochemistry

HepG2 cells were seeded on 15 mm coverslips in 24 well plates (5 × 10^4^ cells/well). After 24 h, cells were treated for further 24 h, fixed with 10% formalin for 15 min and permeabilised with 0.1% Triton X-100. Blocking buffer (5% goat serum PBS) was added for 2 h on a rocker at room temperature. Phalloidin masking was used for staining of actin filaments. Coverslips were mounted with 10 µL of ProLong™ Gold Antifade Mountant with DAPI (ThermoFisher Scientific, Loughborough, UK). Cell morphology was observed using a Leica DMI3000 B microscope.

### 2.14. Statistical Analysis

Data presented are expressed as mean ± SEM. Statistical analysis was performed using Prism 9 software. Normal distributed data sets were subjected to one-way ANOVA with Tukey’s post hoc test for multiple comparison between three or more groups, or to the student’s *t*-test for two groups comparison. Non-normal distributed datasets were subjected to the non-parametric test Kruscal–Wallis with Dunn’s multiple comparison between three or more groups, or to the Mann–Whitney U test for two groups comparison. Sample size (n) represents the number of independent experiments for in vitro studies, or biological replicates for the in vivo study. The degrees of significance are indicated as * *p* ≤ 0.05, ** *p* ≤ 0.01, *** *p* ≤ 0.001, **** *p* ≤ 0.0001.

## 3. Results

### 3.1. PDc Inhibits Proliferation, Adhesion, Migration, and Invasion of HepG2 Cells

**Figure 1:**
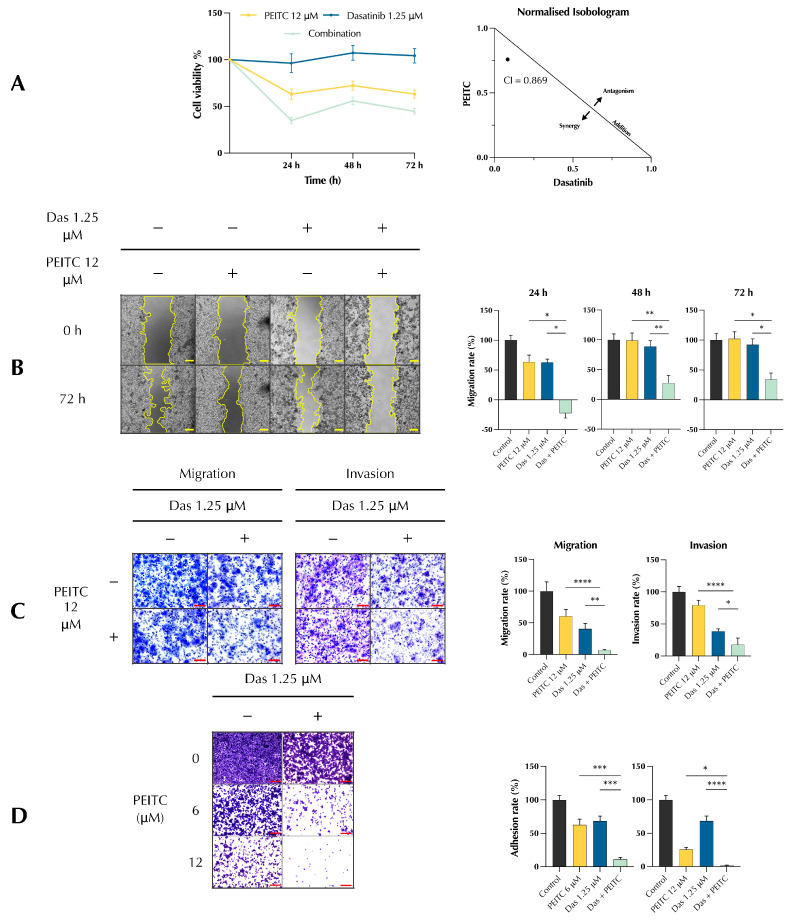
Effect of PDc on cell proliferation, adhesion, migration, and invasion. (**A**) Left, HepG2 cell viability measurement via MTT assay (mean ± SEM, *n* = 3). Right, combination index value at 24 h calculated with CompuSyn software. (**B**) Scratch wound healing assay, representative images (5×, scale bar 200 µm) on the left, quantification of migration rate at 24 h, 48 h, 72 h employing ImageJ 1.55k plugin on the right (mean ± SEM, *n* = 3). (**C**) Migration and invasion assay using Boyden chambers. Left, representative images (20×, scale bar 100 µm); right, quantification of migration and invasion rate (mean ± SEM, *n* = 3). (**D**) Adhesion assay, representative images (10×, scale bar 200 µm) on the left, quantification of adhesion rate by ImageJ 1.55k on the right (mean ± SEM, *n =* 3). The degrees of significance are indicated as * *p* < 0.05, ** *p* < 0.01, *** *p* < 0.001, **** *p* < 0.0001, calculated with one-way ANOVA and Tukey’s post hoc multiple comparisons test.

HepG2 cells were treated with either PEITC (12 μM), dasatinib (1.25 μM), or their combination, and cell viability was measured after 24, 48, and 72 h. The results showed a decrease in cell viability in the combination treatment (Figure 1A Left). To assess the relevance of the drug combination, the Chou–Thalalay method was used to calculate the combination index. Values below 1.0 indicates a synergy, whereas values above 1.0 indicates an antagonism effect. The normalised isobologram (Figure 1A Right) showed a value of 0.869, indicating that the drugs synergise when combined. A scratch wound healing assay determined the ability of PDc to inhibit the migration of HepG2. PDc treatment reduced the ability of the cells to migrate to fill the scratched space (Figure 1B). Next, to assess the PDc effect on migration and invasion, HepG2 cells were subjected to the Boyden chamber assay with or without a coating of extra cellular matrix. The results confirmed the findings from the scratch assay, with a reduction in migrated cells towards the lower chamber (Figure 1C). Furthermore, PDc reduced the ability of HepG2 cells to digest the ECM coating, inhibiting the cell invasive capacity (Figure 1C). Finally, to determine whether PDc can reduce the ability of cancerous cells to adhere to a new ECM, an adhesion assay was employed. After three hours, cell adhesion was strongly impaired by combination treatment, either using PEITC 6 μM or PEITC 12 μM (Figure 1D).

### 3.2. PDc Inhibits Tumour-Induced Adhesion, Migration, and Angiogenesis In Vitro

**Figure 2:**
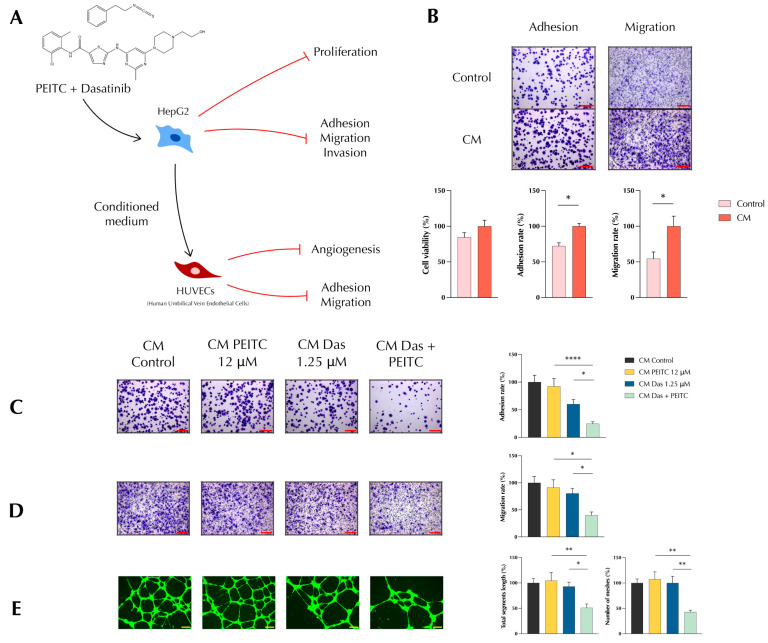
Effect of PDc on tumour-induced angiogenesis in vitro. (**A**) Diagram of HepG2-conditioned medium (CM) model. (**B**) Effect of HepG2-conditioned medium on HUVECs adhesion and migration, representative images (10×, scale bar 20 µm) on the top; quantification of cell viability, adhesion, and migration rate with ImageJ 1.55k on the bottom. Effect of PDc HepG2-conditioned medium on HUVECs: (**C**) adhesion, representative images (10×, scale bar 20 µm) on the left, quantification of adhesion rate using ImageJ 1.55k on the right (mean ± SEM, *n* = 4); (**D**) migration, representative images (10×, scale bar 20 µm) on the left, quantification of migration rate with ImageJ (mean ± SEM, *n* = 4); (**E**) in vitro angiogenesis representative images (4×, scale bar 200 µm) on the left; quantification of segment length and number of meshes with ImageJ on the right (mean ± SEM, *n* = 4). The degrees of significance are indicated as * *p* < 0.05, ** *p* < 0.01, **** *p* < 0.0001 calculated with one-way ANOVA and Tukey’s post hoc multiple comparison.

To assess whether PDc can impinge on the effect that cancerous cells exert on endothelial cells to enhance angiogenesis, HepG2 conditioned medium (CM), with or without PDc treatment, was isolated and used in different HUVECs assays (Figure 2A). HepG2 CM stimulated the proliferation of HUVECs in the MTT assay; in addition, HepG2 CM induced HUVEC’s adhesion and migration in adhesion and Boyden chamber assays, respectively (Figure 2B). The CM isolated after PDc treatment inhibited the induced adhesion and migration (Figure 2C,D), reducing the influence of the cancerous cells on the endothelial cells. Furthermore, PDc was able to inhibit the formation of new vessels in vitro. Indeed, the tube formation assay showed that PDc CM impaired the ability of HUVECs to form a network of vessels, reducing the total length of segments and the total number of meshes (Figure 2E).

### 3.3. PDc Inhibits the Secretion of Pro-Angiogenic Factors

**Figure 3:**
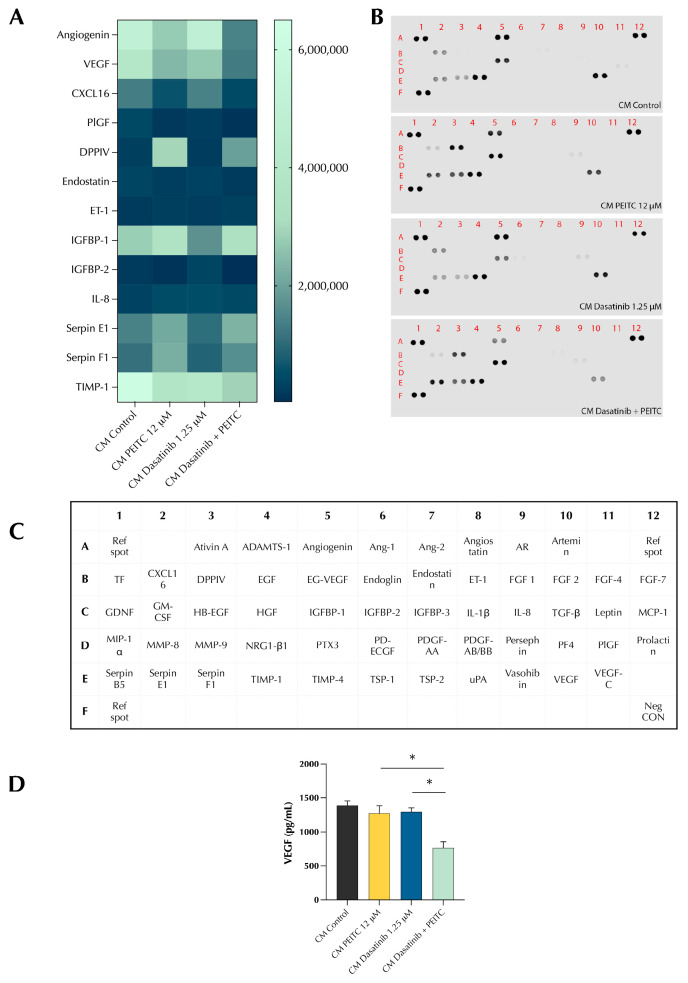
Effect of PDc on HepG2-conditioned medium. (**A**) Heat map of angiogenesis proteome profiler. (**B**) Blots of Angiogenesis Proteome Profiler. (**C**) Legend of targets location on blot. (**D**) VEGF quantification of conditioned medium via ELISA. The degrees of significance are indicated as * *p* < 0.05, calculated with one-way ANOVA and Tukey’s post hoc multiple comparison.

The results of HUVECs assays uncovered the ability of PDc to affect the influence of cancerous cell on endothelial cells, reducing their ability to recruit and promote new vessel formation. To determine what factors are influenced by PDc treatment, CM was analysed using an Angiogenesis Proteome Profiler (Figure 3A–C). PDc treatment reduced the secretion of pro-angiogenic factors. In particular, the combination treatment reduced the concentration of VEGF and angiogenin, and to a lesser extent the concentration of CXCL16 in CM (Figure 3A). Furthermore, the expression of TIMP-1 resulted as lower in the CM of PDc-treated cells, whilst the expression of endostatin resulted similarly low in PDc- and PEITC-treated cells. Moreover, the expression of Serpin E1 resulted as slightly increased in PDc and PEITC CM compared with the control and dasatinib. To confirm the results of the proteome profiler, an ELISA was employed to precisely quantify VEGF in CM. The results showed that PDc reduced the amount of VEGF in CM by 50% (Figure 3D).

### 3.4. PDc Reduces Tumour-Induced Angiogenesis In Vivo

**Figure 4:**
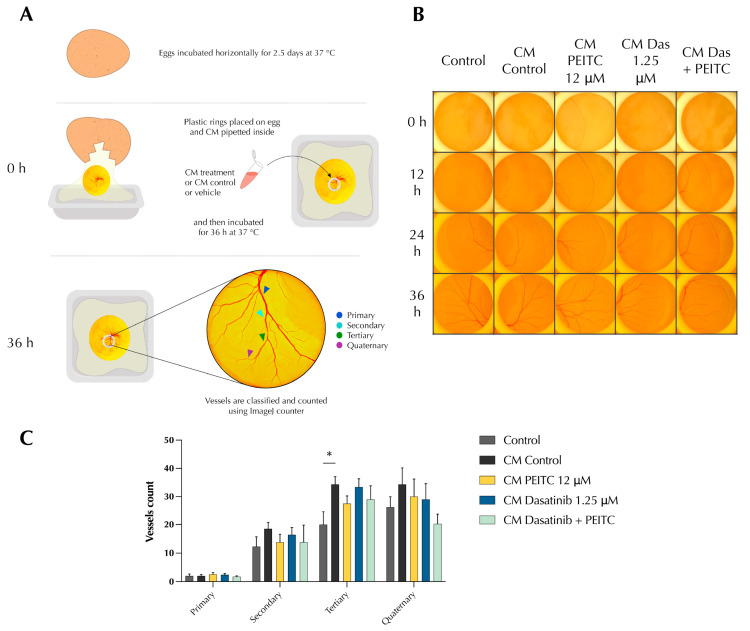
Effect of PDc on HepG2-CM-induced angiogenesis in vivo. (**A**) Diagram of HepG2-conditioned medium model yolk sac membrane assay. (**B**) Representative images of YSM assay from three independent experiments (100×, scale bar 20 µm). (**C**) Quantification based on vessel size hierarchy using ImageJ 1.55k from three independent experiments (mean ± SEM, Control *n* = 4, CM Control *n* = 6, CM PEITC *n* = 4, CM Dasatinib *n* = 5, CM P + D *n* = 4). The degrees of significance are indicated as * *p* < 0.05, calculated with Student’s *t*-test.

The effect of PDc on tumour-induced angiogenesis was evaluated in vivo using a chick yolk membrane (YSM) ex ovo assay. The yolk sac membrane is highly vascularised to allow the delivery of nutrients to the developing chick. Thus, the application of a solution can give insight into the ability of that solution to promote or inhibit angiogenesis. CM (2.5 mg/mL) was applied to the yolk sac membrane with a plastic ring and then pictures were taken every 12 h for 36 h, when the vessels develop to a complexity that allow the observation of quaternary vessels; these are the smallest vessels that originated from the branches, which, in turn, arise from branches of a principal vessel (Figure 4A,B). The results showed that CM from HepG2 stimulated the formation of new vessels. Treatment with CM PDc showed a trend in reducing the number of quaternary vessels (Figure 4C).

### 3.5. PDc Inhibits EMT through FAK/STAT3 Signalling

**Figure 5:**
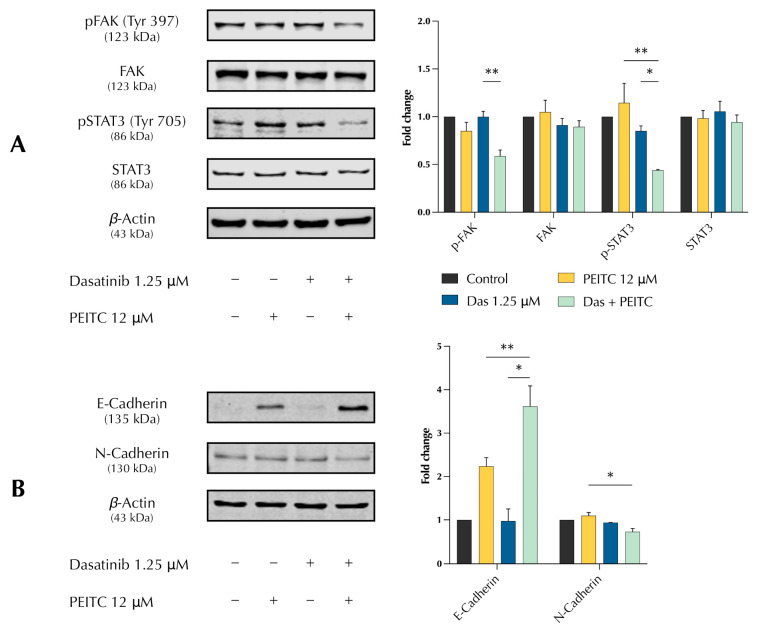
Effect of PDc FAK/STAT3/Cadherin signalling (**A**) Western blot analysis of FAK and STAT3 expression; images are representative of three independent protein extractions. Data are depicted as fold change to control normalised to β-Actin expression (mean ± SEM, *n* ≥ 3) (**B**) Western blot analysis of E-Cadherin and N-Cadherin expression; images are representative of three independent protein extractions. Data are depicted as fold change to control normalised to β-Actin expression (mean ± SEM, *n* ≥ 3). The degrees of significance are indicated as * *p* < 0.05, ** *p* < 0.01, calculated using one-way ANOVA and Tukey’s post hoc multiple comparison.

Considering that dasatinib efficacy is limited by FAK activation and that ITCs are known to inhibit FAK, it was hypothesised that FAK plays a central role in the synergistic anticancer activity of PDc. Focal adhesions have a fundamental role in linking the cytoskeleton with the ECM, allowing the cells to migrate and receive external stimuli. Focal adhesions contain multiple components, including FAK, which communicate with structural proteins, such as the cadherin family members. FAK has a role in numerous cellular processes such as proliferation, migration, and invasion. Protein expression analysis using Western blot confirmed that PDc reduced the activation of FAK (Figure 5A). Furthermore, PDc treatment reduced the activation of STAT3, which is involved in the activation of transcription factors for the expression of genes involved in proliferation, migration, invasion, and angiogenesis (Figure 5A). Finally, to confirm the effect of PDc on the migratory capability of HepG2 cells, protein expression analysis showed a marked increase in E-cadherin expression and a slight decrease in N-cadherin Figure 5B).

### 3.6. PDc Deeply Changes Cytoskeleton Morphology of HepG2 Cells

**Figure 6:**
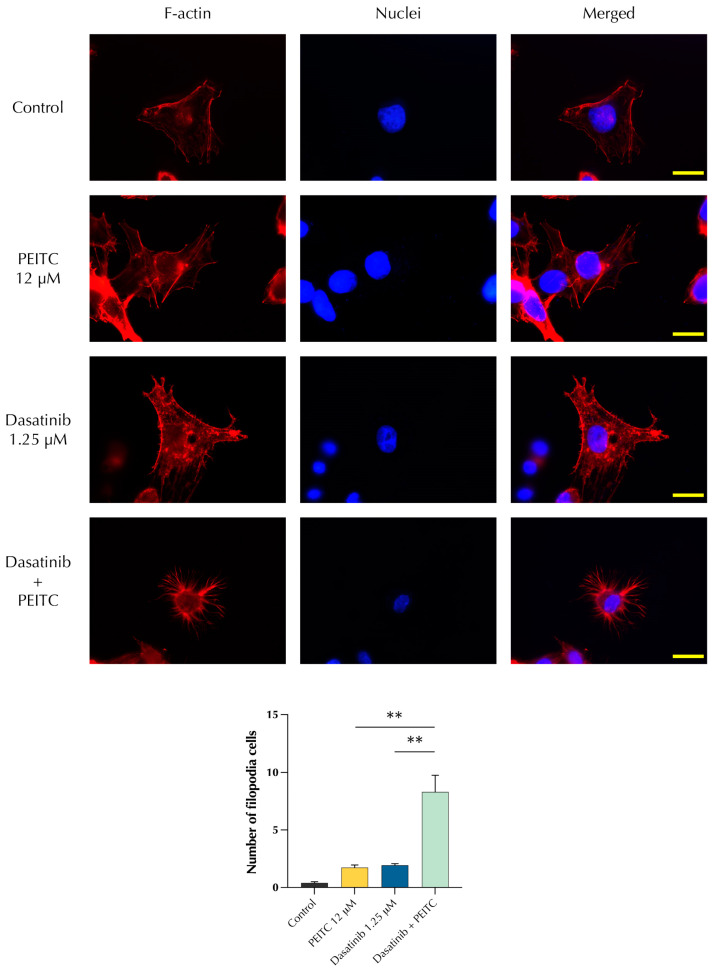
Effect of PDc on HepG2 morphology. Top, phalloidin staining of f-actin in red, DAPI staining of nuclei in blue. Bottom, quantification of cells presenting filopodia structures. The degrees of significance are indicated as ** *p* < 0.01, calculated using one-way ANOVA and Tukey’s post hoc multiple comparison.

The actin cytoskeleton plays a fundamental role in the adhesion to the ECM and it is physically linked to focal adhesion complexes. Furthermore, adhesion is a starting point for the activation of downstream pathways such as the FAK/STAT3 signalling axes [23]. The effect of PDc on cytoskeleton organisation was assessed 1 h after PDc treatment using fluorescence microscopy with a phalloidin staining that binds to f-actin. The phalloidin staining of HepG2 showed that the drug combination induced changes in the organisation of actin cytoskeleton. Usually, cells form wide lamellipodia to interact with the ECM, as observed in the control or in single drug-treated cells (Figure 6). PDc-treated cells showed a different morphology, with cells often shrunk and presenting numerous thin filopodia. Quantification confirmed that the combination treatment increased the number of cells presenting filopodia compared with the control and the single drug treatment.

### 3.7. PDc Inhibits the Growth of a HCC Xenograft

**Figure 7:**
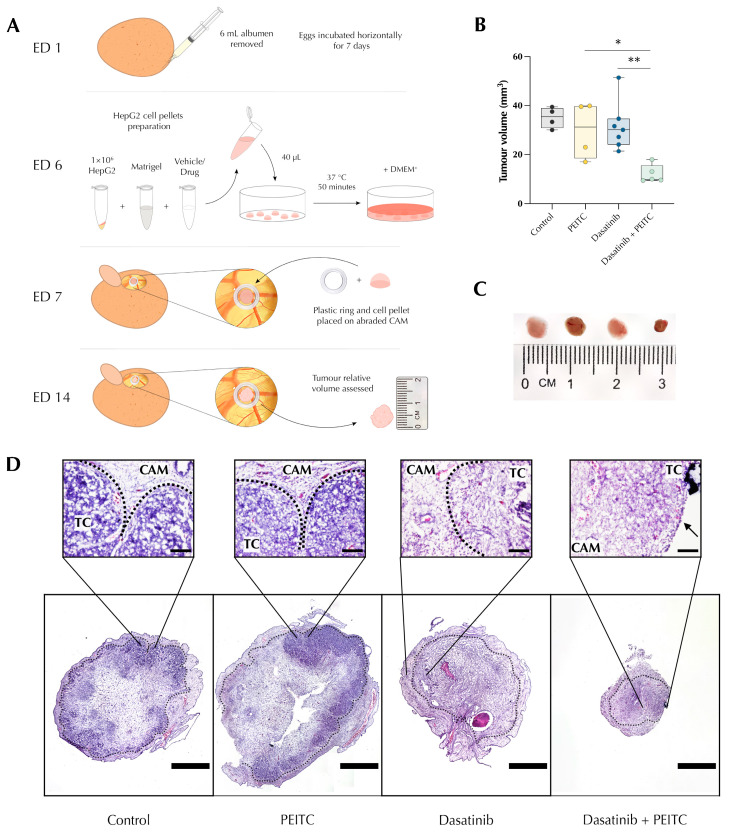
Effect of PDc on HepG2 chorioallantoic membrane (CAM) xenograft. (**A**) Diagram of HepG2 CAM xenograft method. (**B**) Quantification of tumour volume in three independent experiments (mean ± SEM, Control *n* = 4, PEITC *n* = 4, Dasatinib *n* = 6, P + D *n* = 5). The degrees of significance are indicated as * *p* < 0.05, ** *p* < 0.01, calculated using one-way ANOVA and Tukey’s post hoc multiple comparison. (**C**) Representative images of resected HepG2 xenografts (from the left: control, PEITC, dasatinib, PDc). (**D**) CAM xenograft histology H&E staining. TC = tumour cells, CAM = chorioallantoic membrane, dotted lines separation of tumour cells and CAM. Arrow indicate the edge of the tumour mass that is not surrounded by the CAM. Scale bar = 1 mm.

The effect of PDc on the ability of HepG2 cells to adhere and invade an extracellular environment in vivo was tested with a xenograft in ovo chorioallantoic membrane (CAM) assay. A 3D culture of HepG2 cells using an ECM gel treated with either a single drug or their combination was prepared and placed on top of the CAM (Figure 7A). Once the tumour cells are in contact with the CAM, they invade the membrane and recruit endothelial cells to promote angiogenesis, allowing the growth of a tumour mass. After 14 days, the mass is excised and the tumour is measured to assess the ability of PDc to inhibit the HepG2 metastatic potential. The results showed that the combination treatment inhibited the growth of HCC xenografts to a reduced volume (Figure 7B,C). A histological analysis of the resected tumours highlighted the difference of tumour embedding in the CAM (Figure 7D). The H&E-stained control and single drug-treated xenografts were completely embedded in the CAM with invaginations that indicate the expansion of the xenograft into the CAM. On the other hand, combination-treated xenografts were not integrated into the CAM membrane, with the margin indicated by the arrow completely free from the interaction with the CAM (Figure 7D). It appears that the PDc-treated xenografts were unable to invade the vascularised membrane, inhibiting the retrieval of nutrients and explaining the reduced growth.

## 4. Discussion

In this study, we describe synergism between the anti-cancer drug dasatinib and the dietary isothiocyanate PEITC. PDc impaired the ability of HepG2 cells to migrate, invade, and adhere, which are the prerequisites for tumour metastatic dissemination. Furthermore, PDc inhibited the secretion of pro-angiogenic factors, reducing angiogenesis induced by tumour cells. In addition, the drug combination was effective in halting the growth of HCC xenografts in a CAM assay.

Dasatinib is an approved chemotherapeutic agent that showed strong cytotoxicity towards numerous HCC cell lines, but its potency is limited in vivo by the activation of FAK [7]. One of the major targets of dasatinib is the c-Src tyrosine kinase, which phosphorylates FAK, leading to its activation and thus, the formation of new focal adhesion complexes and the activation of downstream signalling pathways involved in cell survival, proliferation, and migration [24,25]. FAK has been linked to the uncontrolled proliferation and migration of tumour cells and being over-expressed in different types of tumours [26]. In the work of Liu et al. [7], one of the main conclusions was that the combination of dasatinib with FAK inhibitors may improve dasatinib potency in HCC. This was corroborated when dasatinib was shown to synergise with rosuvastatin in HCC, and the combined improved anti-cancer activity was associated with the inhibition of the Src/FAK pathway [10]. ITCs are known to inhibit FAK signalling and PEITC is the most effective FAK inhibitor [27]. In this study, protein expression analysis showed that PDc significantly reduced the phosphorylation of FAK at Tyr-397. This residue binds c-Src, leading to the enzymatic activation that propagates the downstream signal [26,28]. Focal adhesion complexes, induced by c-src and FAK activity, stabilise actin filaments. [29]. Phalloidin staining of cytoskeletal actin filaments shortly after treatment with PDc highlighted that the drug combination leads to shrunk morphology and the formation of numerous filopodia. Filopodia are extended structures with sensory functions that reorganise into lamellipodia, allowing cell adhesion and migration [30]. The increase in filopodia formation has been observed before in breast cancer cells treated with resveratrol [30] where it was accompanied by the inhibition of cell migration, and a reduction in focal adhesion complexes and Tyr-397 FAK phosphorylation. In a different study, FAK was differentially activated in adherent and non-adherent cells and its inactivation led to the loss of cell adhesion and cell death [23]. It can be hypothesised that one of the effects of PDc is the inhibition of focal adhesion complexes that leads to the observed loss of adhesion, resulting in cell death.

FAK is also involved in the process of epithelial–mesenchymal transition (EMT), which is a normal developmental program activated during embryogenesis [31]. Tumour cells acquire metastatic capabilities through the hijacking of the EMT. FAK has been previously linked to the JAK/STAT3 pathway, promoting cell motility and cellular signalling in glioblastoma cells [32], whilst the inhibition of STAT3 repressed the invasive capabilities of astrocytoma cells [33]. Moreover, c-Src/FAK/STAT3 signalling regulates the expression of E-cadherin, controlling the migratory capability of melanoma cells [34]. We found that PDc inhibited STAT3 activation and increased the expression of E-cadherin, thus reducing EMT. During EMT, cells reduce E-cadherin expression and increase the expression of N-cadherin to acquire migratory and invasive capability. Indeed, the so-called “cadherin switch” has been associated with breast cancer progression [35]. A lower expression of E-cadherin is often associated with more invasive tumours, and for this reason, E-cadherin is considered a tumour suppressor [36]. Carcinoma cells such as HCC have a peculiar way of increasing motility and invasiveness, defined as “partial EMT”. These cancerous cells are able to gain mesenchymal traits and retain epithelial ones [37]. Therefore, carcinoma cells can migrate and invade new tissues in a collective migration, keeping cell–cell interactions [38]. PDc inhibited both the mesenchymal and epithelial traits of HepG2 cells, reducing migratory and adhesive capabilities at the same time. Hence, the efficacy of this drug combination may rely on the ability to specifically target the partial EMT that characterises HCC cells.

Angiogenesis plays a fundamental role in tumour metastasis; in fact, cancerous cells require new vessels to extravasate into the bloodstream to escape the primary site and reach other body regions to induce the formation of a new network of vessels [39]. In normal conditions, angiogenesis is a process restricted to embryogenesis and is only turned on transiently in fully developed humans [40]. In tumour progression, de novo formation of vessels is required to sustain the growth of a tumour mass. Therefore, cancerous cells control the expression of angiogenic factors to recruit endothelial cells and drive the sprouting of new vessels [40]. Several anti-angiogenic drugs have been developed, but their efficacy was not consistent. After an initial positive effect, the growth of the tumour is usually re-established, probably due to the development of tumour drug resistance [41]. A strategy to tackle tumour drug resistance was introduced with the concept of metronomic therapy, which uses low and frequent doses of chemotherapeutic agents [42]. The association of multiple drugs at low doses could make metronomic therapy even more effective [43]. Dasatinib displayed positive results when associated with angiogenesis inhibitors in breast cancer and HCC [44,45], while ITCs inhibited angiogenesis in several tumour models [46,47,48]. Specifically, PEITC suppressed the expression of VEGF through HIF-1α and VEGFR2 downregulation [49,50,51]. We show here that conditioned medium obtained from HepG2 cells treated with PDc reduced the ability of HUVECs to adhere, migrate, and organise into tubular structures in vitro. In addition, the number of quaternary vessels was reduced after the application of CM to the YSM, a trend which may become significant with more concentrated CM.

The analysis of the CM content using an Angiogenesis Proteome Profiler showed that PDc treatment of HepG2 influenced different proteins that have controversial roles (endostatin, Serpin E1, TIMP-1). Collagen XVIII is a precursor of endostatin, which is considered a potential inhibitor of angiogenesis with therapeutic relevance [52], but its role in HCC remains unclear [53]. Indeed, several reports have shown that the increased expression of endostatin/collagen XVIII is correlated with high levels of VEGF in HCC patients and this was associated with a poor prognosis [52,54]. Therefore, PDc may exert further anti-angiogenic effects with the reduction in endostatin secretion from HCC cells, which can also be associated with the reduction in VEGF expression. Different published works indicate SERPINE1 as an overexpressed gene in poor prognosis HCC patients [55,56], but there is a lack of reports regarding the level of Serpin E1 protein in HCC. Interestingly, Nossin et al. showed that Serpin E1 possess anti-angiogenic properties in mesenchymal stem cells [57]. TIMP-1, as an inhibitor of metalloproteinases, is generally referred to as an anti-angiogenic factor [58,59]. Contrastingly, other reports have identified a different and opposite role for TIMP-1, depending on the stage of the angiogenic process [60]. Indeed, high serum levels of TIMP-1 were found in patients with liver metastasis; hence, TIMP-1 is recognised as negative prognostic factor [61]. Song et al. identified TIMP-1 as a potential target in HCC as it activates the invasion and metastasis of tumour cells, inhibiting tumour apoptosis [62]. High levels of serum TIMP-1 were correlated with increased levels of VEGF in patients with liver metastasis [63]. Therefore, PDc may also contribute to the decrease in another factor involved in a poorer prognosis for HCC patients.

Interestingly, the Angiogenesis Proteome Profiler showed that PDc reduced the secretion of several pro-angiogenic factors (angiogenin, CXCL16, VEGF). This could explain the effects observed in HUVECs and YSM treated with CM. High levels of angiogenin in serum have been associated with poor prognosis in different tumours, and with higher tumour vascularity and the activation of hepatic stellate cells in HCC [64,65,66]. Angiogenin expression has been associated with STAT3 signalling in prostate cancer [67] and with HIF-1α in oral cancer [68]. CXCL16 was identified as an angiogenesis mediator [69], able to attract and stimulate HUVECs proliferation, migration, and the formation of new vessel networks [70,71]. Furthermore, CXCL16 is associated with the increased secretion of VEGF [72] and its expression is promoted in hypoxic conditions through the activation of HIF-1α [71]. VEGF is a critical pro-angiogenic factor highly expressed in tumour development [73] through the over-activation of STAT3 [74] that, in turn, activates gene expression mediated by HIF-1α [75]. Indeed, we showed previously that the inhibition of STAT3/HIF-1α signalling by the ITC sulforaphane reduced angiogenesis in HepG2 cells [76]. Finally, STAT3 control of angiogenesis is associated with c-Src/FAK regulation in ER+ breast cancer cells [77]. Therefore, the PDc-mediated reduction of pro-angiogenic factor secretion can be associated with the reduced activation of FAK/STAT3 pathway.

## 5. Conclusions

It can be hypothesised that the PDc synergistic effect is due to the multiple targeting of c-Src/FAK signalling, affecting actin cytoskeleton organisation, EMT through STAT3 E-cadherin expression, and angiogenesis through the STAT3/HIF-1α control of pro-angiogenic factors. Future work is needed to further investigate the involvement of other pathways and molecular targets responsible for the combination synergistic effects, and the use of patient-derived samples may better predict patient’s treatment responses. Furthermore, the efficacy and safety of PDc have to be validated in more complex organisms (i.e., murine models) to assess the dose and administration modality to achieve the safest result in the clinical setting. Taken together, the results presented in this work indicate that ITCs combined with clinically approved chemotherapeutic agents may represent a valid therapy solution to improve the life expectancy of HCC patients.

## Data Availability

No datasets were generated or analysed during this study and all data are available upon request from the corresponding author.

## Supplementary Materials

The following supporting information can be downloaded at https://www.mdpi.com/article/10.3390/pharmaceutics15102390/s1, Figure S1: Representative full blots of FAK and p-FAK (Tyr397); Figure S2: Representative full blots of STAT3 and p-STAT3 (Tyr705); Figure S3: Representative full blots of E-cadherin; Figure S4: Representative full blots of N-cadherin; Figure S5: Dose-response curves of PEITC and dasatinib at 24 h in HepG2; Figure S6: PDc suppresses viability, adhesion and migration of Hepa 1-6 murine cell line; Table S1: Key resources; Table S2: List of antibodies used in Western blot; Table S3: IC50 values of PEITC and dasatinib at 24 h in HepG2.
