# Supplementary material for: Combination of Phenethyl Isothiocyanate and Dasatinib Inhibits Hepatocellular Carcinoma Metastatic Potential through FAK/STAT3/Cadherin Signalling and Reduction of VEGF Secretion
Source: Pharmaceutics. 2023 Sep 27;15(10):2390. doi: 10.3390/pharmaceutics15102390 (PMC10610226; doi:10.3390/pharmaceutics15102390)
Supplement: Supplementary file 1 [file pharmaceutics-15-02390-s001.zip › pharmaceutics-2599563-supplementary.pdf]

Table S1 | Key resources

Product	Supplier	Identifier
**Materials**		
ThinCert™ 24 well 8 µm	Greiner Bio-One, Kremsmünster, Austria	662638
Amicon® Ultra-15 3 kDa	Merck Life Science, Gillingham, UK	UFC900396
Leukosilk tape	Health Care Equipment and Supplies Ltd, Ipswich, UK	HCP1309
Round glass coverslips 15 mm	Scientific Laboratory Supplies, Nottingham, UK	MIC3338
Square glass coverslips 18 mm	Chance Propper Ltd., West Midlands, UK	N/A
Teflon rings	Eastern Seals, Ashington, UK	BS013PTFE
MX35 Ultra low profile blades	Epredia, Portsmouth, New Hampshire, USA	3053835
Plastic cryomoulds	Agar Scientific Ltd, Stansted, UK	AGG4580
Menzel-Glaser Superfrost plus slides	ThermoFisher Scientific, Loughborough, UK	J1810AMNZ
**Chemicals**		
DMEM	Gibco, ThermoFisher Scientific, Loughborough, UK	10938-025
MEM	Gibco, ThermoFisher Scientific, Loughborough, UK	32561029
FBS	Gibco, ThermoFisher Scientific, Loughborough, UK	10500-064
L-glutamine (200 mM)	Gibco, ThermoFisher Scientific, Loughborough, UK	25030081
Penicillin 100 U/ml Streptomycin (100 µg/ml)	Gibco, ThermoFisher Scientific, Loughborough, UK	15070-063
Trypsin EDTA 0.25%, 0.05%	Gibco, ThermoFisher Scientific, Loughborough, UK	25200-056
Goat serum	Merck Life Science, Gillingham, UK	G9023
Matrigel®	Corning, Somerville, Massachusetts, USA	354230
Cultrex Basement Membrane Extract, Type 3	Bio-Techne Ltd, Abingdon, UK	3632-005-02
PEITC	Merck Life Science, Gillingham, UK	253731
Dasatinib	Merck Life Science, Gillingham, UK	SML2589
**Staining and histology**		
Crystal violet	Merck Life Science, Gillingham, UK	V5265

Product	Supplier	Identifier
Calcein AM	Merck Life Science, Gillingham, UK	C1359
Rhodamine Phalloidin	ThermoFisher Scientific, Loughborough, UK	R415
ProLong™ Gold Antifade Mountant with DAPI	ThermoFisher Scientific, Loughborough, UK	P36931
OCT compound	Agar Scientific Ltd, Stansted, UK	AGR1180
Haematoxylin	Merck Life Science, Gillingham, UK	HHS32
Eosin	Merck KGaA, Darmstadt, Germany	HX884852
Shandon Xylene substitute	ThermoFisher Scientific, Loughborough, UK	FIS9999122
DPX mountant	VWR, Radnor, Pennsylvania, USA	MERC1.00579.0500_P
**Commercial assays**		
Angiogenesis Human Proteome Profiler™	Bio-Techne Ltd, Abingdon, UK	ARY007
DuoSet® ELISA Ancillary kit	Bio-Techne Ltd, Abingdon, UK	DY008
DuoSet® ELISA VEGF kit	Bio-Techne Ltd, Abingdon, UK	DY293B
**Western blotting**		
Bradford reagent	Merck Life Science, Gillingham, UK	B6916
Protease Inhibitor Cocktail (100X)	Cell Signaling Technologies, Leiden, NL	5871S
Pierce™ Phosphatase Inhibitor Mini Tablets	ThermoFisher Scientific, Loughborough, UK	A32957
4x NuPage LDS sample buffer	Invitrogen, ThermoFisher Scientific, Loughborough, UK	NP0007
TruPAGE™ TEA-Tricine SDS Running Buffer	Merck Life Science, Gillingham, UK	PCG30001
PageRuler™ Plus	Invitrogen, ThermoFisher Scientific, Loughborough, UK	26619<br>26616
Immobilon FL PVDF	Merck Life Science, Gillingham, UK	IPFL00010
TBS blocking buffer	LI-COR Biotechnology, Cambridge, UK	927-60001
IRDye® 800CW Streptavidin	LI-COR Biotechnology, Cambridge, UK	926-32230
**Experimental models: cell lines and organisms**		
HepG2 cell line	American Type Culture Collection (ATCC), Manassas, Virginia, USA	HB-8065
HUVECs cell line	PromoCell GmbH, Heidelberg, Germany	C-12200
Fertilised Shaver Brown chicken eggs	Henry Stewart & Co Ltd, UK	

Product	Supplier	Identifier
Hepa 1-6	American Type Culture Collection (ATCC), Manassas, Virginia, USA	CRL-1830
**CRITICAL INSTRUMENTS**		
EVOS M5000 Imaging System	ThermoFisher Scientific, Loughborough, UK	N/A
Leica DMI3000 B	Leica Biosystems Nussloch GmbH, Nußloch, Germany	N/A
FLUOstar Omega	BMG Labtech Ltd., Bucks, UK	N/A
Odyssey CLx Imaging System	LI-COR Biotechnology, Cambridge, UK	N/A
Mini Protean Tank	Bio-Rad Laboratories, Hercules, California, USA	N/A
Trans-Blot® Turbo™ Transfer System	Bio-Rad Laboratories, Hercules, California, USA	N/A
Jasco FP-920	Oklahoma City, Oklahoma, USA	N/A
Leica CM1850 UV	Leica Biosystems Nussloch GmbH, Nußloch, Germany	N/A
**SOFTWARE AND ALGORITHMS**		
GraphPad Prism 9	GraphPad software, San Diego, California USA	v9.1.1
Compusyn	ComboSyn Inc., Paramus, New Jersey USA	N/A
Image Studio Lite 5.2.5	LI-COR Biotechnology, Cambridge, UK	v5.2.5
ImageJ 1.53k	National Institute of Health, Bethesda, Maryland, USA	v1.53k

TABLE S2 | List of antibodies used in Western blot

	Antigen	Host	Dilution	Molecular weight	Supplier	Catalogue number
**Primary antibodies**	FAK	Mouse	1:200 (1 μg/mL)	125 kDa	Santa Cruz Biotech	sc-1688
	p-FAK (Tyr397)	Mouse	1:200 (1 μg/mL)	125 kDa	Santa Cruz Biotech	sc-81493
	STAT3	Rabbit	1:2000 (0.5 μg/mL)	79, 86 kDa	Upstate	06-596
	p-STAT3 (Tyr705)	Rabbit	1:2000	79, 86 kDa	Cell Signaling Technologies	#9145
	E-Cadherin	Goat	1:4000 (0.025 μg/mL)	135 kDa	R&D Systems	AF748
	N-Cadherin	Mouse	1:2000 (0.25 μg/mL)	130 kDa	R&D Systems	MAB13881
	β-Actin	Mouse	1:2000 (0.1 μg/mL)	43 kDa	Santa Cruz Biotech	sc-47778
	β-Actin	Rabbit	1:5000	43 kDa	Cell Signaling Technologies	#4970
**Secondary antibodies**	IRDye® 800CW Mouse	Donkey	1:10000 (1 μg/mL)	N/A	LI-COR	926-32212
	IRDye® 800CW Rabbit	Donkey	1:10000 (1 μg/mL)	N/A	LI-COR	926-32213
	IRDye® 800CW Goat	Donkey	1:10000 (1 μg/mL)	N/A	LI-COR	926-32214
**Secondary antibodies**	IRDye® 680LT Rabbit	Donkey	1:10000 (1 μg/mL)	N/A	LI-COR	926-68023
	IRDye® 680LT Mouse	Goat	1:10000 (1 μg/mL)	N/A	LI-COR	926-68020

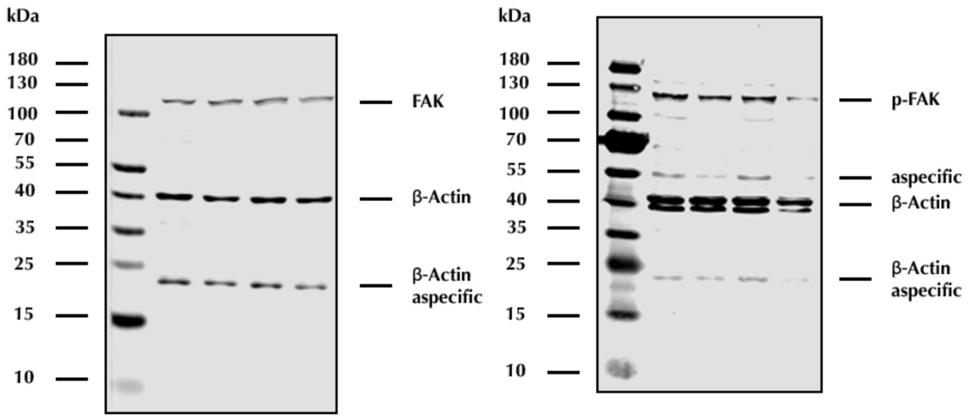

FIGURE S1 | Representative full blots of FAK and p-FAK (Tyr397).
Samples order: Control, PEITC 12, dasatinib 1.25, PEITC + dasatinib.

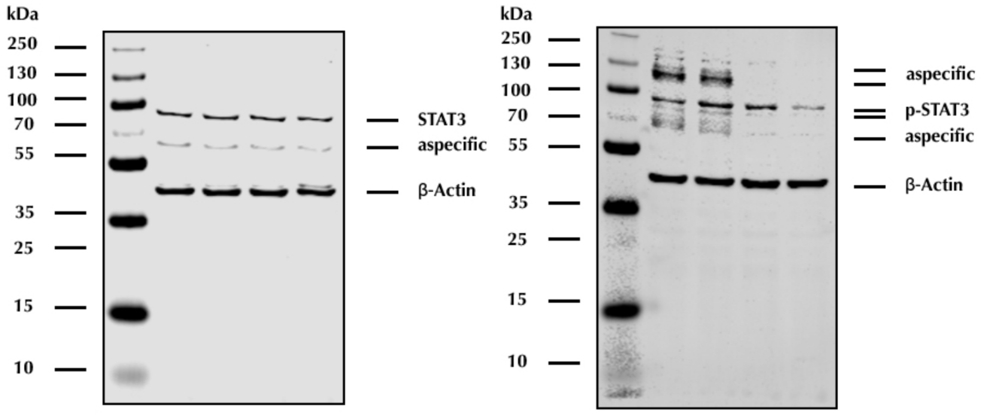

FIGURE S2 | Representative full blots of STAT3 and p-STAT3 (Tyr705).
Samples order: Control, PEITC 12 μM, dasatinib 1.25 μM, PEITC + dasatinib.

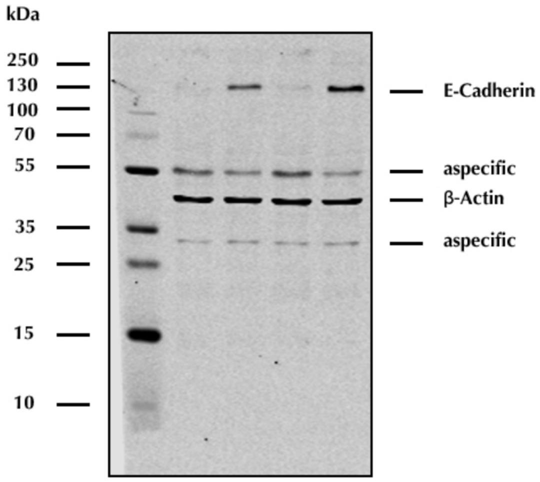

FIGURE S3 | Representative full blots of E-cadherin.
Samples order: Control, PEITC 12 μM, dasatinib 1.25 μM, PEITC + dasatinib.

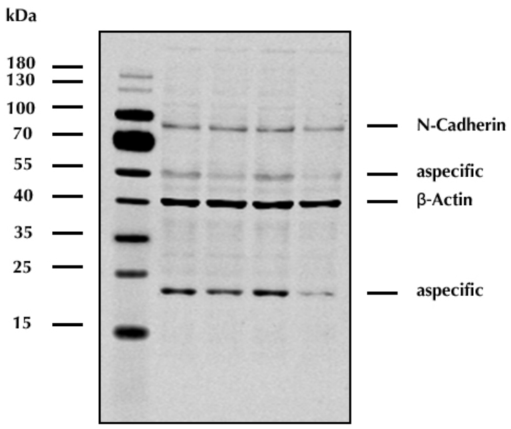

FIGURE S4 | Representative full blots of N-cadherin.
Samples order: Control, PEITC 12 μM, dasatinib 1.25 μM, PEITC + dasatinib.

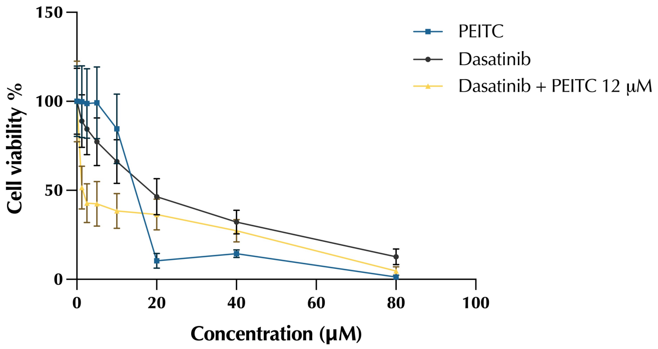

FIGURE S5 | Dose-response curves of PEITC and dasatinib at 24 h in HepG2.

TABLE S3 | IC_{50} values of PEITC and dasatinib at 24 h in HepG2.

	IC_{50} (μM)
**PEITC**	15.8
**Dasatinib**	15

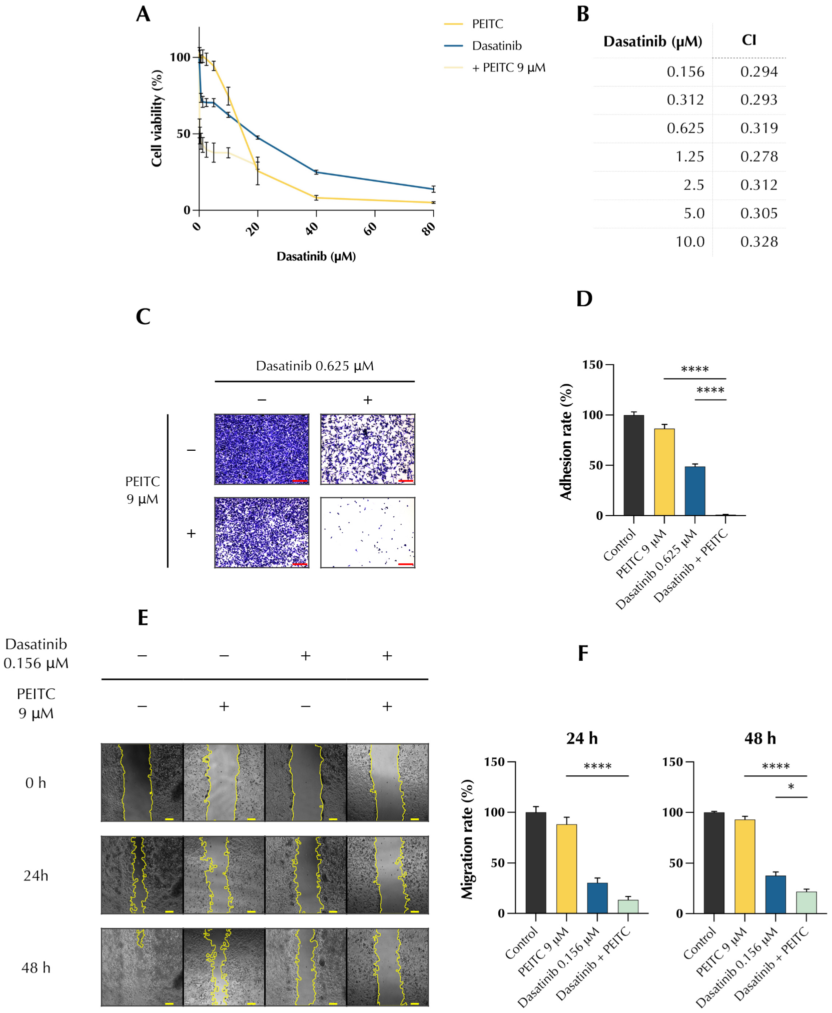

Dasatinib (µM)	CI
0.156	0.294
0.312	0.293
0.625	0.319
1.25	0.278
2.5	0.312
5.0	0.305
10.0	0.328

FIGURE S6 | PDc suppresses viability, adhesion and migration of Hepa 1-6 murine cell line. (**A**) Hepa 1-6 cells were treated with different concentrations of PEITC and dasatinib (0.156 to 10 µM) or with dasatinib plus fixed concentrations of PEITC (6, 9, 12 µM) for 24 h. The data presented were normalised to control's proliferation (mean ± SEM, n=3). (**B**) CI values calculated with CompuSyn using the Chou Talalay method. **(C)** Hepa 1-6 cells were seeded on a 200 µg/mL Matrigel-coated plate and treated with dasatinib 0.625 µM with or without PEITC 9 µM. Cells were stained with crystal violet and imaged with an inverted microscope. Representative images from 3 independent experiments (20x, scale bar 100 µm). **(D)** Adhesion assay's quantitative data were normalised to control (mean ± SEM, n=3). **(E-F)** Hepa 1-6 cells were treated with dasatinib 0.156 µM with or without PEITC 9 µM, and pictures were taken at 0, 24 and 48 h. **(E)** Representative images at 0 h and 72 h. **(F)** Quantitative data presented were normalised to control's migration rate (mean ± SEM, n=3). The degrees of significance are indicated as * $p < 0.05$, **** $p < 0.0001$, calculated using one-way ANOVA and Tukey's post hoc multiple comparison.
